# Differential Communications between Fungi and Host Plants Revealed by Secretome Analysis of Phylogenetically Related Endophytic and Pathogenic Fungi

**DOI:** 10.1371/journal.pone.0163368

**Published:** 2016-09-22

**Authors:** Xihui Xu, Qin He, Chen Chen, Chulong Zhang

**Affiliations:** 1 College of Life Sciences, Nanjing Agricultural University, Nanjing, 210095, People’s Republic of China; 2 State Key Laboratory of Rice Biology, Institute of Biotechnology, Zhejiang University, Hangzhou, 310058, People’s Republic of China; 3 Newe Ya'ar Research Center, Agricultural Research Organization, Ramat Yishay, 30095, Israel; Fujian Agriculture and Forestry University, CHINA

## Abstract

During infection, both phytopathogenic and endophytic fungi form intimate contact with living plant cells, and need to resist or disable host defences and modify host metabolism to adapt to their host. Fungi can achieve these changes by secreting proteins and enzymes. A comprehensive comparison of the secretomes of both endophytic and pathogenic fungi can improve our understanding of the interactions between plants and fungi. Although *Magnaporthe oryzae*, *Gaeumannomyces graminis*, and *M*. *poae* are economically important fungal pathogens, and the related species *Harpophora oryzae* is an endophyte, they evolved from a common pathogenic ancestor. We used a pipeline analysis to predict the *H*. *oryzae*, *M*. *oryzae*, *G*. *graminis*, and *M*. *poae* secretomes and identified 1142, 1370, 1001, and 974 proteins, respectively. Orthologue gene analyses demonstrated that the *M*. *oryzae* secretome evolved more rapidly than those of the other three related species, resulting in many species-specific secreted protein-encoding genes, such as avirulence genes. Functional analyses highlighted the abundance of proteins involved in the breakdown of host plant cell walls and oxidation-reduction processes. We identified three novel motifs in the *H*. and *M*. *oryzae* secretomes, which may play key roles in the interaction between rice and *H*. *oryzae*. Furthermore, we found that expression of the *H*. *oryzae* secretome involved in plant cell wall degradation was downregulated, but the *M*. *oryzae* secretome was upregulated with many more upregulated genes involved in oxidation-reduction processes. The divergent *in planta* expression patterns of the *H*. and *M*. *oryzae* secretomes reveal differences that are associated with mutualistic and pathogenic interactions, respectively.

## Introduction

Fungi play essential roles in diverse environments, including the establishment of various relationships with host plants with interactions that range from mutualistic to pathogenic [1, 2]. During infection, fungi can secrete proteins called effectors to manipulate the immunity and physiology of their hosts to prevent host detection, suppress plant defences, and/or induce plant cell death [3–5]. LysM effectors, a class of conserved effectors that have no recognisable protein domains except for the LysM motif, can interfere with chitin-induced plant immunity and promote infection [6]. The Slp1 protein from *Magnaporthe oryzae* is an example of a LysM effector [7]. In addition, carbohydrate-degrading enzymes may also be secreted by fungi to feed on complex molecules [1].

Similar to phytopathogens, which need to resist or disable host defences and modify host metabolism to adapt to their host [8, 9], endophytic fungi form intimate intercellular contacts with living plant cells, which are usually mutualistic associations [10, 11]. Thus, endophytes also need to manipulate host defences and metabolism. Communication between fungi and host plants is achieved by secretion of proteins and enzymes that are either delivered into the plant cells or detected at the plant cell surface [1, 12]. This communication occurs, in particular, at the initial stage of infection [13]. Recent studies have shown that endophytism is evolutionarily transient, with endophytic lineages frequently transitioning to and from pathogenicity in phylogenetic trees [14]. For example, clavicipitaceous endophytes arose from insect-parasitic ancestors [15], whereas the beneficial endophyte *Harpophora oryzae* originated from a phytopathogenic ancestor [16]. Thus, a comprehensive comparison of the secretomes of closely related endophytic and pathogenic fungi sharing a common ancestor will further our understanding of the interactions between fungi and host plants.

Rice blast, caused by the fungal pathogen *M*. *oryzae*, is one of the most severe rice diseases and it occurs almost everywhere rice is grown [17, 18]. In contrast, the beneficial dark septate endophytes (DSEs) such as *H*. *oryzae*, which resides in domestic Chinese wild rice (*Oryza granulata*), can not only strongly promote rice growth and biomass accumulation [19], but also protect rice roots from invasion by *M*. *oryzae* and induce systemic resistance to rice blast, thus making it an attractive candidate for biocontrol [20]. Phylogenetic analysis revealed that *H*. *oryzae* is closely related to other members of the Magnaporthaceae, such as the plant pathogens *M*. *oryzae*, *Gaeumannomyces graminis*, and *M*. *poae* [19]. A detailed comparative study of *H*. and *M*. *oryzae* at the morphologic, physiological, and genomic levels has revealed that these two species originated from a common pathogenic ancestor and gain or loss of orphan genes, gene family expansions, and transposable element activities were important factors in this evolution [16]. Furthermore, divergent defence responses and carbon allocation patterns have been detected in the interaction of *H*. and *M*. *oryzae* with rice [21], demonstrating that host plants were also involved in this evolution. However, limited information is available about secretomes, which may also play important roles considering the vital function of secreted proteins in the communication between fungi and plants. In this study, we present a comprehensive comparison of the secretomes of the endophyte *H*. *oryzae* and its closely related pathogenic fungi to provide new insights into endophyte- and pathogen-plant interactions.

## Results

### Predicted *H*. *oryzae*, *M*. *oryzae*, *G*. *graminis*, and *M*. *poae* secretomes

**Fig 1:**
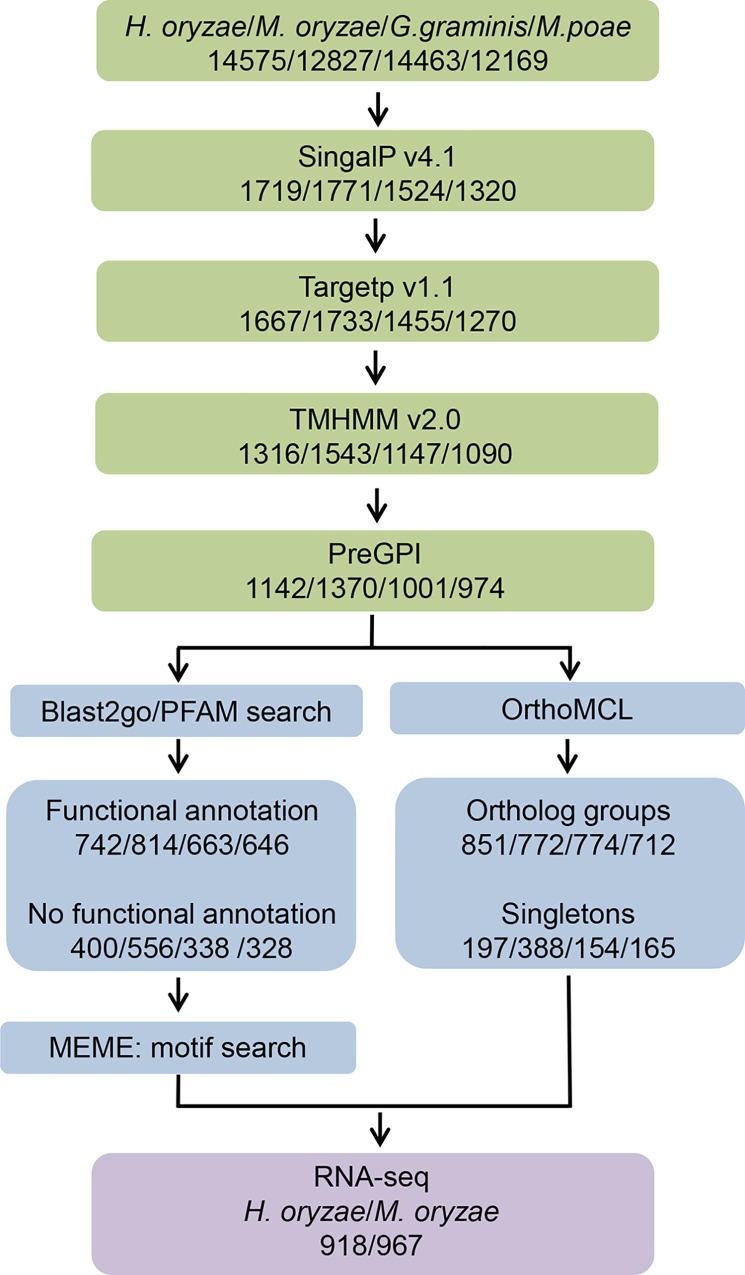
The analysis pipeline applied to the *H*. *oryzae*, *M*. *oryzae*, *G*. *graminis*, and *M*. *poae* secretomes. The pipeline can be divided in three main steps: 1) secretome prediction, 2) functional analysis, and 3) expression analysis.

In total, we detected 1667, 1733, 1455, and 1279 genes encoding secreted proteins in the *H*. *oryzae*, *M*. *oryzae*, *G*. *graminis*, and *M*. *poae* genomes, respectively, representing 11.4, 13.5, 10.1, and 10.4% of the respective genomes (Fig 1). Within these gene sets, proteins predicted to contain transmembrane (TM) domain or glycosylphosphatidylinositol (GPI)-anchored proteins were removed. This resulted in predicted secretomes of 1142, 1370, 1001, and 974 proteins for *H*. *oryzae*, *M*. *oryzae*, *G*. *graminis*, and *M*. *poae*, respectively (7.8, 10.6, 6.9, and 8.0% of total genomes, respectively) (Fig 1; S1 Table).

**Fig 2:**
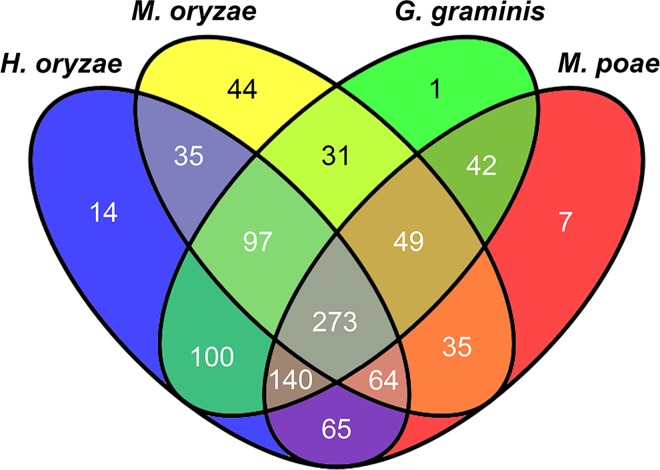
Venn diagram showing orthologues among the *H*. *oryzae*, *M*. *oryzae*, *G*. *graminis*, and *M*. *poae* secretomes. The values indicate the counts of the orthologue groups.

Markov Cluster algorithm (MCL) analysis identified a total of 987 orthologue groups clustered among the predicted *H*. *oryzae*, *M*. *oryzae*, *G*. *graminis*, and *M*. *poae* secretomes, with only 273 orthologue groups shared by all four species (Fig 2). On average, each group contained approximately 3.1 genes. A total of 851 (75%), 772 (56%), 774 (77%), and 712 (73%) secreted proteins of *H*. *oryzae*, *M*. *oryzae*, *G*. *graminis*, and *M*. *poae*, respectively, clustered into MCL groups (S2 Table). Of these, 232 *M*. *oryzae* secreted proteins (distributed in 89 groups) were present in one or more paralogues, considerably more than in *H*. *oryzae* (129 in 59 groups), *G*. *graminis* (68 in 27 groups), and *M*. *poae* (81 in 34 groups). Moreover, we identified 44, 14, 1, and 7 species-specific groups for the four respective secretomes (Fig 2; S2 Table).

### Functional annotation of secretomes

**Fig 3:**
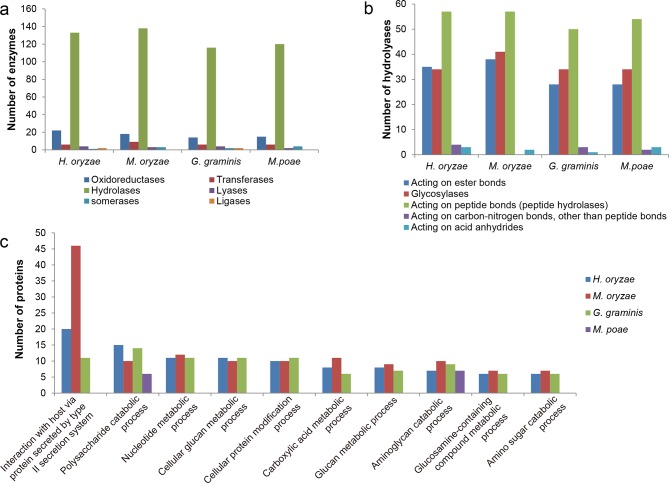
Profiles of *H*. *oryzae*, *M*. *oryzae*, *G*. *graminis*, and *M*. *poae* annotated secretomes. (a) Major enzyme classes in the four secretomes. (b) Major targets of the hydrolyases. (c) The 10 most abundant GO categories (biological processes, level 6) of the four secretomes.

Blast2GO analysis revealed that 400, 556, 338, and 328 secreted proteins had no functional annotation, whereas 742, 814, 663, and 646 secreted proteins, in the *H*. *oryzae*, *M*. *oryzae*, *G*. *graminis*, and *M*. *poae* secretomes, respectively, had some form of annotation such as InterPro, SUPERFAMILY, or PFAM entries (Fig 1; S1 Table). Among the four unannotated secretomes, 197 (49%), 388 (70%), 154 (46%), and 165 (50%) were singletons, which were not included in any orthologue groups. The general enzymatic activities (EC classification) and Gene Ontology (GO) biological process annotations of the annotated secretomes of the four species were involved in some form of catalytic activity, represented primarily by hydrolyases and oxidoreductases (Fig 3; S3 Table). The major targets of the hydrolyases were lipids, carbohydrates, and proteins (Fig 3). To identify the metabolic pathways involved, enzymes from the four secretomes were mapped onto the Kyoto Encyclopedia of Genes and Genomes (KEGG) metabolic pathways (Table 1), showing the high representation of enzymes related to phenylpropanoid biosynthesis, amino/nucleotide sugar metabolism, starch/sucrose metabolism, and aminobenzoate degradation.

**Table 1:** Secreted proteins involved in KEGG metabolic pathways.

KEGG pathway^a^	*H*. *oryzae*		*M*. *oryzae*		*G*. *graminis*		*M*. *poae*	
	Pro^b^	Enz^c^	Pro	Enz	Pro	Enz	Pro	Enz
Phenylpropanoid biosynthesis	15	1	10	1	9	1	10	2
Amino sugar and nucleotide sugar metabolism	13	4	16	4	12	5	12	3
Drug metabolism—other enzymes	11	2	16	1	7	1	8	1
Other glycan degradation	10	5	12	4	7	4	9	5
Starch and sucrose metabolism	9	6	7	3	8	3	10	6
Aminobenzoate degradation	6	2	10	2	5	2	5	2
Glycosphingolipid biosynthesis-globo series	6	3	4	2	5	3	3	3
Pentose and glucuronate interconversions	5	4	5	3	4	2	4	3
Sphingolipid metabolism	4	2	4	3	5	3	2	2
Biosynthesis of antibiotics	3	2	6	4	3	2	4	4
Glycerophospholipid metabolism	3	3	3	2	2	2	3	1
Glycosaminoglycan degradation	3	2	2	1	1	1	1	1
Glyoxylate and dicarboxylate metabolism	3	2	2	1	4	2	2	2
Tryptophan metabolism	3	2	2	1	4	2	2	2
Various types of N-glycan biosynthesis	3	2	6	2	3	2	3	2
Alanine, aspartate and glutamate metabolism	2	2	0	0	1	1	1	1
beta-Alanine metabolism	2	1	2	1	2	1	3	1
Cyanoamino acid metabolism	2	2	2	1	1	1	1	1
Galactose metabolism	2	1	2	1	3	1	1	1
Glutathione metabolism	2	2	2	1	1	1	0	0
Glycerolipid metabolism	2	1	4	2	4	2	2	2
Glycine, serine and threonine metabolism	2	1	4	2	2	1	0	0
Glycosphingolipid biosynthesis—ganglio series	2	1	2	1	1	1	1	1
Glycosphingolipid biosynthesis-lacto and neolacto series	2	1	0	0	1	1	1	1
Isoquinoline alkaloid biosynthesis	2	1	2	1	2	1	3	1
N-Glycan biosynthesis	2	2	6	3	4	2	3	2
Phenylalanine metabolism	2	1	2	1	2	1	3	1
Purine metabolism	2	3	3	4	1	2	4	6
Tropane, piperidine and pyridine alkaloid biosynthesis	2	1	2	1	2	1	3	1
Tyrosine metabolism	2	1	2	1	2	1	3	1

^a^ Only the 30 most abundant pathways are listed.

^b^ Number of proteins

^c^ Number of enzymes.

### Comparative PFAM analysis of secretomes

Of the *H*. *oryzae*, *M*. *oryzae*, *G*. *graminis*, and *M*. *poae* annotated protein sequences, 576, 601, 535, and 497, respectively, contained at least one PFAM domain (S4 Table) and 41 (120 domains), 46 (133 domains), 40 (118 domains), and 40 (117 domains) hydrolase families were identified among 107, 114, 100, and 101 proteins (S5 Table), representing 14, 14, 15, and 16%, respectively, of the four annotated secretomes. GH61 (PF03443, now AA9 family), was one of the most abundant PFAM domains found in 24, 17, 16, and 15 proteins of the *H*. *oryzae*, *M*. *oryzae*, *G*. *graminis*, and *M*. *poae* secretomes, respectively (Table 2). In addition, Peptidase_S8 (PF00082) was also abundant in the four secretomes (Table 2). For carbohydrate-binding modules, WSC (PF01822) and LysM (PF01476) domains were more abundant in the *H*. *oryzae*, *G*. *graminis*, and *M*. *poae* secretomes, whereas the CBM_1 (PF00734) domain was more abundant in the *M*. *oryzae* secretome. Another abundant PFAM domain was a type B carboxylesterase (COesterase, PF00135) implicated in lipid degradation (Table 2). These results suggested the most abundant PFAM domains were hydrolytic enzymes involved in the breakdown of host plant cell walls.

**Table 2:** The 25 most abundant PFAM domains in the four secretomes.

PFAM	PFAM definition	*H*. *oryzae*	*M*. *oryzae*	*G*. *graminis*	*M*. *poae*
PF03443.11	Glyco_hydro_61	24	17	16	15
PF00082.19	Peptidase_S8	18	19	18	13
PF01565.20	FAD_binding_4	18	20	15	10
PF00264.17	Tyrosinase	13	14	15	13
PF08031.9	BBE	13	13	10	7
PF00734.15	CBM_1	12	19	15	8
PF09362.7	DUF1996	12	6	13	12
PF13472.3	Lipase_GDSL_2	12	10	13	6
PF00732.16	GMC_oxred_N	11	12	13	11
PF00026.20	Asp	10	8	9	13
PF00150.15	Cellulase	10	6	8	4
PF04616.11	Glyco_hydro_43	10	12	9	8
PF05199.10	GMC_oxred_C	10	10	13	10
PF00135.25	COesterase	9	14	11	6
PF04389.14	Peptidase_M28	9	11	7	10
PF01083.19	Cutinase	8	11	4	5
PF01476.17	LysM	8	4	8	12
PF07731.11	Cu-oxidase_2	8	10	6	7
PF07732.12	Cu-oxidase_3	8	10	6	8
PF11327.5	DUF3129	8	6	7	7
PF00394.19	Cu-oxidase	7	9	5	7
PF00704.25	Glyco_hydro_18	7	10	9	8
PF01328.14	Peroxidase_2	7	3	5	6
PF01822.16	WSC	7	5	7	6
PF06280.9	fn3_5	7	7	6	5

Beyond the domains potentially involved in plant cell degradation, we also identified numerous proteins involved in oxidation-reduction processes in the four secretomes, including proteins containing FAD_binding_4 (PF01565), GMC_oxred_C (PF05199.10), and GMC_oxred_N (PF00732) domains (Table 2; S5 Table).

### Identification of motifs in unannotated secretomes

**Fig 4:**
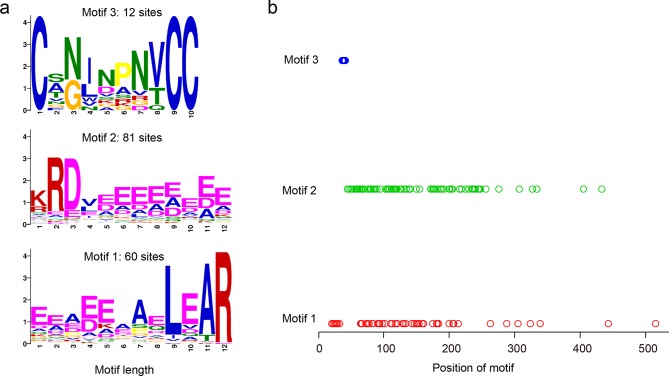
*De novo* motif searches of the unannotated *H*. and *M*. *oryzae* secretomes. (a) Sequences of the three motifs. (b) Distribution of the three motifs in the amino acid sequence of each protein. Each circle represents one motif.

Conserved motifs were detected in the unannotated *H*. and *M*. *oryzae* secretomes. Three constrained motifs were identified, ranging in abundance from 12 to 81 sites (Fig 4). Compared to *M*. *oryzae*, the unannotated *H*. *oryzae* secretome contained far more sites of motif 1 (48 sites) and motif 2 (53 sites), with 27 proteins containing both motifs.

Motif 1 contained L, A, and R residues which were conserved and organised in an LXAR pattern. This motif was identified in 23 orthologue groups. Motif 2 contained R and D residues in an RD pattern; 28 orthologue groups contained proteins with motif 2, and 10 groups shared motif 1. Finally, motif 3 was the least abundant motif, identified in only 12 proteins distributed in 4 groups, but none of the groups overlapped with those of motifs 1 or 2. Motif 4 contained a conserved V/TCC pattern that was preceded by a conserved C residue, located at an average position of 36 amino acids (Fig 4).

### Divergent *in planta* expression patterns of *H*. and *M*. *oryzae* secretomes

**Fig 5:**
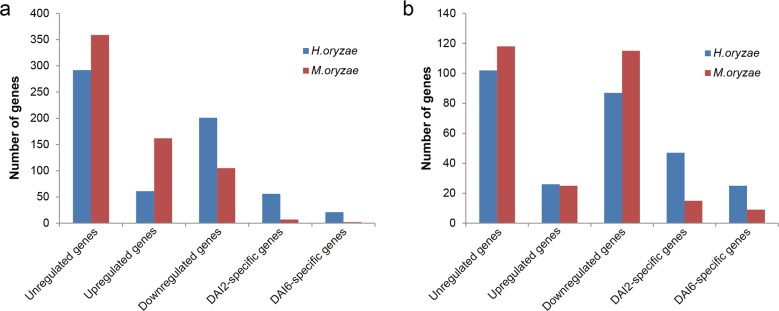
Summary of *H*. and *M*. *oryzae* secretome expression. Fold-changes of gene expression (DAI6 versus DAI2) are presented. (a) Annotated secretomes. (b) Unannotated secretomes. DAI2 and DAI6 refer to transcripts expressed by *H*. or *M*. *oryzae* infecting rice roots at 2 and 6 days after inoculation, respectively. Unregulated genes, genes with fold-change ≤ 2 and ≥ 0.5; Upregulated genes, genes with fold change > 2; Downregulated genes, genes with fold change < 0.5.

Of 1142 predicted members of the *H*. *oryzae* secretome, 918 genes encoding secreted proteins (80%) were expressed at 2 or 6 days after infection (HDAI2 or HDAI6) in rice roots, including 631 annotated and 287 unannotated genes (S6 Table). In total, 967 genes (71%) out of 1370 *M*. *oryzae* secretome genes were expressed at 2 or 6 days after infection (MDAI2 or MDAI6) in rice roots, with 635 annotated and 282 unannotated genes (S6 Table). Although 394 (43%) and 477 (49%) genes showed similar expression levels for DAI2 and DAI6 (fold change < 2), the expression patterns differed between the *H*. and *M*. *oryzae* secretomes during the infection of rice roots (Fig 5; S6 Table). Furthermore, 149 proteins of the *H*. *oryzae* secretome exhibited time-dependent expression (103 for HDAI2 and 46 for HDAI6), a much larger number than for *M*. *oryzae* (22 for MDAI2 and 11 for MDAI2) (Fig 5; S6 Table). Sixty one genes in the annotated *H*. *oryzae* secretome were upregulated during the infection process (DAI6/DAI2 > 2), a markedly smaller number than for *M*. *oryzae* (162 genes) (Fig 5A; S6 Table). At the same time, 201 annotated genes of the *H*. *oryzae* secretome were downregulated (DAI6/DAI2 < 0.5), many more than for the *M*. *oryzae* secretome (105 genes). However, no divergence in expression patterns was found between the unannotated secretomes (Fig 5B; S6 Table).

**Fig 6:**
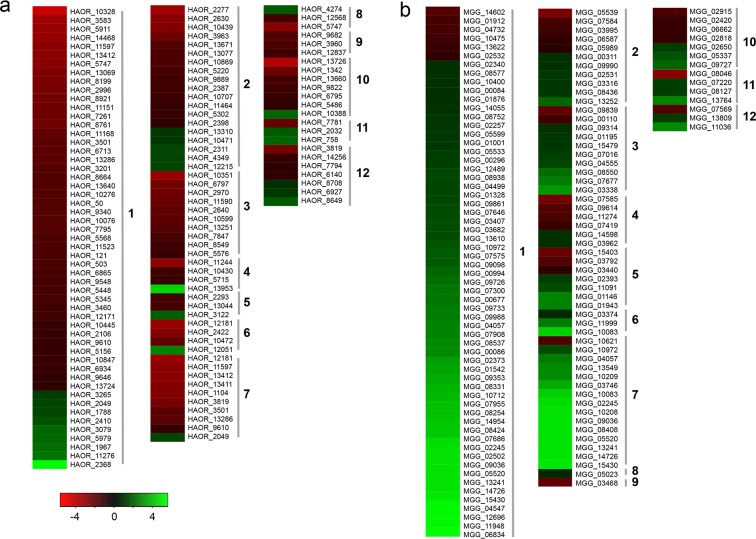
Expression patterns of genes encoding secreted proteins in *H*. and *M*. *oryzae*. (a) *H*. *oryzae* genes. (b) *M*. *oryzae* genes. Red, decrease in transcript abundance; green, increase in transcript abundance. Heat maps were produced based on expression changes (log2 DAI6/DAI2). 1, proteins with Glyco_hydro domain; 2, peptidase; 3, lipase; 4, tyrosinase; 5, cutinase; 6, cellulase; 7, proteins with CBM domain; 8, proteins with Chitin_bind_1 domain; 9, proteins with LysM domain, 10, proteins with FAD_binding_4 domain; 11, proteins with Cu-oxidase domain; and 12, proteins with GMC_oxred_N domain.

We further analysed the functions of the secretome genes that exhibited differential expression levels (Fig 6). Most of the *H*. *oryzae* genes involved in plant cell wall degradation, such as proteins containing Glyco_hydro, peptidase, lipase, tyrosinase, cutinase, cellulase, CBM, and LysM domains, were downregulated during infection. However, most of the genes with the same function were upregulated during the *M*. *oryzae* infection process. In addition, far more genes of the *M*. *oryzae* secretome involved in oxidation-reduction processes (including proteins with FAD_binding, GMC_oxred, and Cu-oxidase domains) were upregulated compared with *H*. *oryzae* (Fig 6).

For the *H*. *oryzae* secretome, we detected expression of 63 genes encoding proteins containing motif 1, 2, or 3, including 26 genes with both motifs 1 and 2. Among these, 24 genes were downregulated, whereas two were upregulated. Fewer genes encoding proteins with motifs 1, 2, or 3 were expressed in *M*. *oryzae* (32 genes), 18 of which were downregulated and two were upregulated (S6 Table).

## Discussion

Promotion and regulation of the infection process is essential for both endophytic and pathogenic fungi. Analysis of the secretome is a powerful tool to investigate how fungi manage the infection process [5, 22]. For example, analysis of the secretomes of *Puccinia graminis* and *Melampsora larici-populina* using an *in silico* comprehensive analysis pipeline identified eight families of candidate effectors with high value for functional characterisation [22]. Investigation of the arsenal of proteins secreted by *Sclerotinia sclerotiorum* and *Botrytis cinerea*, identified refined secretomes with 432 and 499 proteins, respectively, providing a greater understanding of the necrotrophic infection process [5]. In our previous study of the *H*. *oryzae* genome, we focused on the characterisation of 167 small secreted cysteine-rich proteins (SSCRPs), which contained more than 4% cysteine residues and were at most 200 amino acids in length [16]. The prediction of these secreted proteins used a less stringent selection criteria and was based on a limited interspecies comparison, while the expression patterns were almost ignored. Therefore, in this study, the detailed analysis of the secretomes of four closely related fungi, namely *H*. *oryzae*, *M*. *oryzae*, *G*. *graminis*, and *M*. *poae* (including both an endophyte and pathogens) provides us with much more information about how secretomes affect infection by fungi with different lifestyles, and allow more informed choices to be made when choosing secreted proteins to be verified for function during infection.

We predicted full and refined *H*. *oryzae*, *M*. *oryzae*, *G*. *graminis*, and *M*. *poae* secretomes containing 1142, 1370, 1001, and 974 proteins, respectively, thereby significantly expanding the analysis of SSCRPs reported previously [16]. Some secreted protein-encoding genes such as the Avr-Pita1 genes (MGG_15370, MGG_14981, and MGG_15212), which were clustered in an *M*. *oryzae*-specific group, and the Avr-piz gene MGG_18041, which was present as a singleton, may function as avirulence genes in *M*. *oryzae*. Frequent presence/absence of polymorphisms in Avr-genes and rapid evolution of avirulence genes have been detected in *M*. *oryzae* and diverse repeated sequences are closely associated with most Avr-genes [23]. Approximately 75% of proteins from the *H*. *oryzae*, *G*. *graminis*, and *M*. *poae* secretomes were consistently present in orthologue groups, more than those of *M*. *oryzae* (56%). In addition, the number of proteins with paralogues and species-specific groups in *M*. *oryzae* was greater than for the other three species. Furthermore, more singletons were present in the *M*. *oryzae* secretome. These results suggest that the *M*. *oryzae* secretome may have evolved more rapidly than those of the three related species, resulting in many species-specific secreted proteins. This process would have also led to the retention of fewer secreted proteins of the common ancestor in *M*. *oryzae* than in the three other species.

PFAM analysis of the four secretomes revealed the abundance of enzymes involved in the breakdown of host plant cell walls, including hydrolysing enzymes that degrade various plant host substrates. Evaluation of the enzymatic activities, GO biological processes, and KEGG metabolic pathways assigned to the secreted proteins confirmed the high representation of polysaccharide catabolic and proteolytic processes among the four secretomes. GH61, the most abundant PFAM domain, is important for degrading lignocellulose in conjunction with cellulases [24]. Although it is not truly a glycosidase, this family is listed as a glycosyl hydrolase in the CAZy classification [25]. In addition, Peptidase_S8, a family of serine proteases, may contribute to plant cell wall degradation by targeting structural proteins, such as hydroxyproline-rich glycoproteins [26, 27]. LysM domains could bind to chitin, a major constituent of fungal cell walls, which can be recognised by host cell surface receptors and trigger an immune response [28]. To overcome immunity, both endophytes and pathogens secrete effector molecules involved in fungal cell wall modification [29, 30]. Four secreted LysM-containing proteins of *H*. and *M*. *oryzae* are expressed consistently during the infection of rice roots.

Characterisation of the role of reactive oxygen species (ROS) has shown that the redox state of the host substrate is extremely important in disease establishment [31, 32]. At the same time, ROS originating from a mutualistic endophyte are required to inactivate plant defence responses and maintain mutualism [33]. It has been shown that virulent pathogens, such as *M*. *oryzae*, have developed mechanisms for scavenging ROS [34], whereas *H*. *oryzae* possesses melanin as an adaptive countermeasure to tolerate oxidative stress [20]. Consistent with these findings, our functional analysis of the four secretomes revealed a large number of proteins predicted to be involved in oxidation-reduction interactions. Enzymes that use FAD as a co-factor are primarily oxygen-dependent oxidoreductases, whereas GMC oxidoreductases constitute a large protein family with oxidoreductase activity which catalyses the transfer of electrons between molecules. In fungi, these enzymes are involved in many processes, such as defence and virulence mechanisms, browning and pigmentation, and melanin production [35, 36]. In addition, tyrosinases, which are abundant in *H*. *oryzae*, may also be involved in multiple biological processes, including melanin production [35, 36].

Using a *de novo* motif search, we detected only three conserved patterns in the combined unannotated secretomes of *H*. and *M*. *oryzae*, which is much less than was described in a similar study [22]. One possible reason for fewer conserved patterns is the rapid evolution of the *M*. *oryzae* secretome. This explanation is also supported by the fact that many more motif sites were detected in the *H*. *oryzae* secretome than in the *M*. *oryzae* secretome. Furthermore, compared to *M*. *oryzae*, more motif-containing proteins were expressed during the infection process in *H*. *oryzae*. These results suggest that the motif-containing genes may play more important roles for infection by *H*. *oryzae* than *M*. *oryzae*.

The induction of host defences has been reported in the symbiotic interaction of plants and fungi [21, 37]. However, divergent responses of rice to infection by *H*. *oryzae* and *M*. *oryzae* have been identified, including suppressed defence responses in the interaction with *H*. *oryzae*, in contrast with the persistent defence responses induced by *M*. *oryzae* [37]. The expression levels of the *H*. *oryzae* secretome genes involved in plant cell wall degradation were consistently downregulated, but were upregulated in *M*. *oryzae*. The upregulated expression of the *M*. *oryzae* secretome genes involved in oxidation-reduction processes may have also resulted from the continuous defence responses of rice roots given that ROS play a vital role in stress responses, programmed cell death (PCD), and plant defences [32]. It has been shown that the host plays important roles in the evolutionary processes of fungi [16]. Thus, the different secretome expression patterns of *H*. and *M*. *oryzae* may represent differences in the feedback of these two kinds of fungi when they interact with the host plant, suggesting communication between fungi and host plants. These results also highlight how secretion and regulation of the expression of proteins and enzymes are important means of fungi-plant communication.

## Conclusions

In this study, we characterised the full and refined secretomes of the endophytic fungus *H*. *oryzae* and the related phytopathogenic fungi *M*. *oryzae*, *G*. *garminis*, and *M*. *poae*. The secretomes were abundant in proteins involved in the breakdown of host plant cell walls and in oxidation-reduction processes, and exhibited divergent expression patterns during infection of the same host plant by endophytes or pathogens. The next step is to determine whether the observed differences hold for pairs of related mutualists and endophytes in additional host species across the plant kingdom.

## Materials and Methods

### Secretome prediction

The proteomes of *M*. *oryzae* (version 8), *G*. *graminis* (version 1), and *M*. *poae* (version 1) used in this study were downloaded from the Broad Institute (https://www.broadinstitute.org/) and Genbank (accession number: PRJNA13840, PRJNA37931, and PRJNA13840 respectively). The *H*. *oryzae* proteome was the same as that used in our previous report (accession number: PRJNA252809, version 1) [16]. Predictions of signal peptides and cleavage sites were performed using SignalP v4.1 software [38] and sub-cellular targeting was analysed using TargetP v1.1 software [39] with default parameters. Only proteins predicted as being secreted by both methods were considered for further analyses. The proteins were scanned for transmembrane (TM) spanning regions using TMHMM v2.0 software [40] to exclude proteins likely to be retained in the plasma membrane. GPI-anchor proteins were predicted using PreGPI software [41] with default parameters. The final numbers of proteins belonging to the *H*. *oryzae*, *M*. *oryzae*, *G*. *graminis*, and *M*. *poae* secretomes were 1142, 1370, 1001, and 974, respectively.

### Functional annotation

To characterise putative functions of predicted secretomes, we used BLAST2GO software with automated annotation [42]. Protein sequences were compared with the non-redundant sequence database using the BLASTP algorithm with an e-value less than 1.0e–05 recording max. 20 hits. Proteins with significant hits were classified into GO categories by ‘Mapping’. Data from both analyses were merged into the annotation. PFAM domains were mapped onto proteins of the predicted secretomes using the PFAM batch search server [43] with default parameters. Orthologue groups were identified using OrthoMCL v2.0 software [44] with default parameters. First, orthologue, co-orthologue, and inparalogue pairs were identified with OrthoMCL. The pairs and their weights were used to construct an OrthoMCL graph for clustering with the MCL algorithm [45]. *A de novo* protein motif search was performed on the combined unannotated *H*. and *M*. *oryzae* secretomes using MEME software [46]. The program was set to report the 10 most robust motifs of 4–12 amino acids, occurring zero or one time per sequence with an e-value less than 1.0e–02.

### Expression analysis

*H*. and *M*. *oryzae* RNA-seq data were the same as sets used in our previous study [16], which described the transcriptomes of the two fungi at 2 and 6 days after infection (DAI) of rice roots. Briefly, the blast-susceptible rice cultivar CO-39 (*Oryzae sativa*), the *M*. *oryzae* strain Guy11 and *H*. *oryzae* strain R5-6-1 were used in the experiment. Rice seeds were surface sterilized as previously described [20] and planted in half-strength Murashige & Skoog solid medium at 30°C in the dark for 4 days and then grown vertically under a 16-h light/8-h dark photoperiod at 28/24°C for an additional 6 days. *M*. *oryzae* were cultured under a 16-h light/8-h dark photoperiod at 25°C, while *H*. *oryzae* were cultured in the dark at 25°C. Conidia of *M*. *oryzae* were harvested from 10-day-old cultures grown on solid complete medium, and germinating phialidic conidia of *H*. *oryzae* were harvested from 4-day-old potato dextrose broth. Inoculations and co-culturing were performed as described previously [16]. All samples were harvested and immediately flash frozen in liquid nitrogen. Total RNA was extracted and then sequenced using the Illumina HiSeq 2000 based on 100bp paired-end read sequencing [16]. All the clean reads were mapped to the genome sequences using TopHat v 2.0.9 [47], and an expression profile was created using Cufflinks v2.0.2 [48]. The RNA-seq data were also validated by quantitative real time (qRT)-PCR, the results of which showed high consistency [16]. Gene expression levels were quantified by fragments per kilobase of exon per million fragments (FPKM) analysis and genes with FPKM values greater than one were considered to be expressed. Heat maps of gene expression profiles were generated using R (www.R-project.org) based on expression changes (log2-fold changes). All data were checked to avoid problems related to Excel converting gene identifiers [49].

## Supporting Information

S1 Table Predicted *H*. *oryzae*, *M*. *oryzae*, *G*. *graminis*, and *M*. *poae* secretomes. (XLSX)

S2 Table Summary of orthologue groups in the *H*. *oryzae* secretome compared to the three related species. (XLSX)

S3 Table GO distribution of biological processes by level (6). (XLSX)

S4 Table PFAM analyses of the *H*. *oryzae*, *M*. *oryzae*, *G*. *graminis*, and *M*. *poae* secretomes. (XLSX)

S5 Table Summary of PFAM domains in *H*. *oryzae*, *M*. *oryzae*, *G*. *graminis*, and *M*. *poae* secretomes. (XLSX)

S6 Table Expression of genes encoding secreted proteins in the *H*. and *M*. oryzae secretomes. (XLSX)

## References

[1] Brown JK, Tellier A. Plant-parasite coevolution: bridging the gap between genetics and ecology. Annu Rev Phytopathol. 2011; 49: 345–367. 10.1146/annurev-phyto-072910-095301 21513455

[2] Eaton CJ, Cox MP, Scott B. What triggers grass endophytes to switch from mutualism to pathogenism?. Plant Sci. 2011; 180(2): 190–195. 10.1016/j.plantsci.2010.10.002 21421360

[3] Jones JDG, Dangl JL. The plant immune system. Nature. 2006; 444(7117): 323–329. 17108957 10.1038/nature05286

[4] Ökmen B, Doehlemann G. Inside plant. biotrophic strategies to modulate host immunity and metabolism. Curr Opin Plant Biol. 2014; 20: 19–25. 10.1016/j.pbi.2014.03.011 24780462

[5] Heard S, Brown NA, Hammond-Kosack K. An interspecies comparative analysis of the predicted secretomes of the necrotrophic plant pathogens *Sclerotinia sclerotiorum* and *Botrytis cinerea*. PloS one. 2015; 10(6): e0130534 10.1371/journal.pone.0130534 26107498 PMC4480369

[6] de Jonge R, Thomma BP. Fungal LysM effectors: extinguishers of host immunity?. Trends Microbiol. 2009; 17(4): 151–157. 10.1016/j.tim.2009.01.002 19299132

[7] Mentlak TA, Kombrink A, Shinya T, Ryder LS, Otomo I, Saitoh H, et al Effector-mediated suppression of chitin-triggered immunity by *Magnaporthe oryzae* is necessary for rice blast disease. Plant Cell. 2012; 24(1): 322–335. 10.1105/tpc.111.092957 22267486 PMC3289562

[8] Ellis JG, Rafiqi M, Gan P, Chakrabarti A, Dodds PN. Recent progress in discovery and functional analysis of effector proteins of fungal and oomycete plant pathogens. Curr Opin Plant Biol. 2009; 12: 399–405. 10.1016/j.pbi.2009.05.004 19540152

[9] Koeck M, Hardham AR, Dodds PN. The role of effectors of biotrophic and hemibiotrophic fungi in infection. Cell Microbiol. 2011; 13(12): 1849–1857. 10.1111/j.1462-5822.2011.01665.x 21848815 PMC3218205

[10] Pumplin N, Harrison MJ. Live-cell imaging reveals periarbuscular membrane domains and organelle location in *Medicago truncatula* roots during arbuscular mycorrhizal symbiosis. Plant Physiol. 2009; 151: 809–819. 10.1104/pp.109.141879 19692536 PMC2754618

[11] Rafiqi M, Ellis JG, Ludowici VA, Hardham AR, Dodds PN. Challenges and progress towards understanding the role of effectors in plant-fungal interactions. Curr Opin Plant Biol. 2012; 15(4): 477–482. 10.1016/j.pbi.2012.05.003 22658704

[12] Giraldo MC, Dagdas YF, Gupta YK, Mentlak TA, Yi M, Martinez-Rocha AL, et al Two distinct secretion systems facilitate tissue invasion by the rice blast fungus *Magnaporthe oryzae*. Nat Commun. 2013; 4: 1996 10.1038/ncomms2996 23774898 PMC3709508

[13] Dodds PN, Rathjen JP. Plant immunity: towards an integrated view of plant-pathogen interactions. Nat Rev Genet. 2010; 11(8): 539–548. 10.1038/nrg2812 20585331

[14] Arnold AE, Miadlikowska J, Higgins KL, Sarvate SD, Gugger P, Way A, et al A phylogenetic estimation of trophic transition networks for ascomycetous fungi: are lichens cradles of symbiotrophic fungal diversification?. Syst Biol. 2009; 58(3): 283–297. 10.1093/sysbio/syp001 20525584

[15] Spatafora JW, Sung GH, Sung JM, Hywel-Jones NL, White JF Jr. Phylogenetic evidence for an animal pathogen origin of ergot and the grass endophytes. Mol Ecol. 2007; 16(8): 1701–1711. 17402984 10.1111/j.1365-294X.2007.03225.x

[16] Xu XH, Su ZZ, Wang C, Kubicek CP, Feng XX, Mao LJ, et al The rice endophyte *Harpophora oryzae* genome reveals evolution from a pathogen to a mutualistic endophyte. Sci Rep. 2014; 4: 5783 10.1038/srep05783 25048173 PMC4105740

[17] Talbot NJ. On the trail of a cereal killer: exploring the biology of *Magnaporthe grisea*. Annu Rev Microbiol. 2003; 57: 177–202. 14527276 10.1146/annurev.micro.57.030502.090957

[18] Ebbole DJ. *Magnaporthe* as a model for understanding host-pathogen interactions. Annu Rev Phytopathol. 2007; 45: 437–456. 17489691 10.1146/annurev.phyto.45.062806.094346

[19] Yuan ZL, Lin FC, Zhang CL, Kubicek CP. A new species of *Harpophora* (Magnaporthaceae) recovered from healthy wild rice (*Oryza granulata*) roots, representing a novel member of a beneficial dark septate endophyte. FEMS Microbiol Lett. 2010; 307(1): 94–101. 10.1111/j.1574-6968.2010.01963.x 20402786

[20] Su ZZ, Mao LJ, Li N, Feng XX, Yuan ZL, Wang LW, et al Evidence for biotrophic lifestyle and biocontrol potential of dark deptate endophyte *Harpophora oryzae* to rice blast disease. PloS One. 2013; 8(4): e61332 10.1371/journal.pone.0061332 23637814 PMC3630206

[21] Xu XH, Wang C, Li SX, Su ZZ, Zhou HN, Mao LJ, et al Friend or foe: differential responses of rice to invasion by mutualistic or pathogenic fungi revealed by RNAseq and metabolite profiling. Sci Rep. 2015; 5: 13624 10.1038/srep13624 26346313 PMC4642567

[22] Saunders DG, Win J, Cano LM, Szabo LJ, Kamoun S, Raffaele S. Using hierarchical clustering of secreted protein families to classify and rank candidate effectors of rust fungi. PLoS One. 2012; 7(1): e29847 10.1371/journal.pone.0029847 22238666 PMC3253089

[23] Huang J, Si W, Deng Q, Li P, Yang S. Rapid evolution of avirulence genes in rice blast fungus *Magnaporthe oryzae*. BMC Genet. 2014; 15(1): 1. 24725999 10.1186/1471-2156-15-45 PMC4021558

[24] Langston JA, Shaghasi T, Abbate E, Xu F, Vlasenko E, Sweeney MD. Oxidoreductive cellulose depolymerization by the enzymes cellobiose dehydrogenase and glycoside hydrolase 61. Appl Environ Microbiol. 2011; 77(19): 7007–7015. 10.1128/AEM.05815-11 21821740 PMC3187118

[25] Cantarel BL, Coutinho PM, Rancurel C, Bernard T, Lombard V, Henrissat B. The Carbohydrate-Active EnZymes database (CAZy): an expert resource for Glycogenomics. Nucleic Acids Res. 2009; 37(Database issue): D233–D238. 10.1093/nar/gkn663 18838391 PMC2686590

[26] Dunaevsky YE, Matveeva AR, Beliakova GA, Domash VI, Belozersky MA. Extracellular alkaline proteinase of *Colletotrichum gloeosporioides*. Biochemistry (Mosc). 2007; 72(3): 345–350. 17447890 10.1134/s0006297907030145

[27] Dow JM, Davies HA, Daniels MJ. A metalloprotease from *Xanthomonas campestris* that specifically degrades proline/hydroxyproline-rich glycoproteins of the plant extracellular matrix. Mol Plant Microbe Interact. 1998; 11(11): 1085–1093. 9805395 10.1094/MPMI.1998.11.11.1085

[28] Kombrink A1, Sánchez-Vallet A, Thomma BP. The role of chitin detection in plant-pathogen interactions. Microbes Infect. 2011; 13(14–15): 1168–1176. 10.1016/j.micinf.2011.07.010 21856436

[29] Jones JD, Dangl JL. The plant immune system. Nature. 2006; 444(7117): 323–329. 17108957 10.1038/nature05286

[30] Kombrink A, Thomma BP. LysM effectors: secreted proteins supporting fungal life. PLoS Pathog. 2013; 9(12): e1003769 10.1371/journal.ppat.1003769 24348247 PMC3861536

[31] Williams B, Kabbage M, Kim HJ, Britt R, Dickman MB. Tipping the balance: *Sclerotinia sclerotiorum* secreted oxalic acid suppresses host defenses by manipulating the host redox environment. PLoS Pathog. 2011; 7(6): e1002107 10.1371/journal.ppat.1002107 21738471 PMC3128121

[32] Hamilton CE, Gundel PE, Helander M, Saikkonen K. Endophytic mediation of reactive oxygen species and antioxidant activity in plants: a review. Fungal Divers. 2012; 54(1): 1–10.

[33] Tanaka A, Christensen MJ, Takemoto D, Park P, Scott B. Reactive oxygen species play a role in regulating a fungus-perennial ryegrass mutualistic interaction. Plant Cell. 2006; 18(4): 1052–1066. 16517760 10.1105/tpc.105.039263 PMC1425850

[34] Chi MH, Park SY, Kim S, Lee YH. A novel pathogenicity gene is required in the rice blast fungus to suppress the basal defenses of the host. PLoS Pathog. 2009; 5(4): e1000401 10.1371/journal.ppat.1000401 19390617 PMC2668191

[35] Halaouli S, Asther M, Sigoillot JC, Hamdi M, Lomascolo A. Fungal tyrosinases: new prospects in molecular characteristics, bioengineering and biotechnological applications. J Appl Microbiol. 2006; 100(2): 219–232. 16430498 10.1111/j.1365-2672.2006.02866.x

[36] SolerRivas C, Arpin N, Olivier JM, Wichers HJ. Activation of tyrosinase in *Agaricus bisporus* strains following infection by *Pseudomonas tolaasii* or treatment with a tolaasin-containing preparation. Mycol Res. 1997; 101(3): 375–382.

[37] Liu J, Blaylock LA, Endre G, Cho J, Town CD, VandenBosch KA, et al Transcript profiling coupled with spatial expression analyses reveals genes involved in distinct developmental stages of an arbuscular mycorrhizal symbiosis. Plant Cell. 2003; 15(9): 2106–2123. 12953114 10.1105/tpc.014183 PMC181334

[38] Petersen TN, Brunak S, von Heijne G, Nielsen H. SignalP 4.0: discriminating signal peptides from transmembrane regions. Nat Methods. 2011; 8(10): 785–786. 10.1038/nmeth.1701 21959131

[39] Emanuelsson O, Brunak S, von Heijne G, Nielsen H. Locating proteins in the cell using TargetP, SignalP and related tools. Nat Protoc. 2007; 2(4): 953–971. 17446895 10.1038/nprot.2007.131

[40] Krogh A, Larsson B, von Heijne G, Sonnhammer EL. Predicting transmembrane protein topology with a hidden Markov model: application to complete genomes. J Mol Biol. 2001; 305(3): 567–580. 11152613 10.1006/jmbi.2000.4315

[41] Pierleoni A, Martelli P, Casadio R. PredGPI: a GPI-anchor predictor. BMC bioinformatics. 2008; 9: 392 10.1186/1471-2105-9-392 18811934 PMC2571997

[42] Conesa A, Götz S, García-Gómez JM, Terol J, Talón M, Robles M. Blast2GO: a universal tool for annotation, visualization and analysis in functional genomics research. Bioinformatics. 2005; 21(18): 3674–3676. 16081474 10.1093/bioinformatics/bti610

[43] Finn RD, Mistry J, Tate J, Coggill P, Heger A, Pollington JE, et al The Pfam protein families database. Nucleic Acids Res. 2010; 38 (Database issue): D211–222. 10.1093/nar/gkp985 19920124 PMC2808889

[44] Chen F, Mackey AJ, Stoeckert CJ Jr, Roos DS. OrthoMCL-DB: querying a comprehensive multi-species collection of ortholog groups. Nucleic Acids Res. 2006; 34(Database issue): D363–D368. 16381887 10.1093/nar/gkj123 PMC1347485

[45] Enright AJ, Van Dongen S, Ouzounis CA. An efficient algorithm for large-scale detection of protein families. Nucleic Acids Res. 2002; 30(7): 1575–1584. 11917018 10.1093/nar/30.7.1575 PMC101833

[46] Bailey TL, Boden M, Buske FA, Frith M, Grant CE, Clementi L, et al MEME SUITE: tools for motif discovery and searching. Nucleic Acids Res. 2009; 37(Web Server issue): W202–W208. 10.1093/nar/gkp335 19458158 PMC2703892

[47] Trapnell C, Pachter L, Salzberg SL. TopHat: discovering splice junctions with RNA-Seq. Bioinformatics. 2009; 25(9): 1105–1111. 10.1093/bioinformatics/btp120 19289445 PMC2672628

[48] Trapnell C, Williams BA, Pertea G, Mortazavi A, Kwan G, van Baren MJ, et al Transcript assembly and quantification by RNA-Seq reveals unannotated transcripts and isoform switching during cell differentiation. Nat Biotechnol. 2010; 28(5): 511–515. 10.1038/nbt.1621 20436464 PMC3146043

[49] Ziemann M, Eren Y, El-Osta A. Gene name errors are widespread in the scientific literature. Genome Biol. 2016; 17(1): 177 10.1186/s13059-016-1044-7 27552985 PMC4994289

